# Now We’re Cooking with Gas: Unintended Consequences of Clean Cookstoves

**DOI:** 10.4269/ajtmh.22-0124

**Published:** 2022-03-14

**Authors:** Ned Walker

**Affiliations:** Department of Microbiology and Molecular Genetics, Michigan State University, East Lansing, Michigan

When asked why he robbed banks, the notorious American criminal John Dillinger famously replied: “Because that’s where the money is.” If we were to ask *Anopheles* mosquitoes why they enter houses in Africa, they might similarly reply “because that is where the human blood is.”

Malaria in Africa is a decidedly domestic problem. The species of *Anopheles* that transmit *Plasmodium falciparum* in sub-Saharan Africa, including *Anopheles gambiae*, *Anopheles coluzzii*, *Anopheles arabiensis*, and *Anopheles funestus*, all have strong predilections for the human living environment. Males and females commonly enter and rest in houses, a behavioral phenomenon called endophily; and females bite people indoors at night to obtain blood, ^1^ a phenomenon called anthropophagy. In doing so, they either transmit or acquire malaria parasites. How do these mosquitoes find houses and enter them? How can we keep mosquitoes out? These simple questions have large consequences, but the behavioral foundations for *Anopheles* attraction to human houses are not well understood.

In this issue, Hennessee et al. ^2^ report that cooking with liquified petroleum gas (LPG) in experimental huts in Rwanda increased recruitment of *Anopheles* compared with recruitment in huts where traditional cooking fuels such as wood or charcoal were burned. Although air-borne fine particulates were lower, *Anopheles* survival and host-seeking rates were higher in huts with LPG cooking. The conclusion is that use of LPG to cook results in cleaner air, but increases indoor “mosquito pollution” and, by inference, malaria risk. Liquified petroleum gas generated from locally produced, organic waste for cooking is being promoted in Africa as a clean fuel to reduce the health effects of air pollution, to decrease dependency on dirtier and scarcer fuels, and to enhance a sustainable biogas economy. ^3^^,^ ^4^

Two important conclusions emerge from consideration of the results of Hennessee et al. ^2^ First, large-scale initiatives such as use of LPG fuel to promote cleaner cooking practices in sub-Saharan Africa, however well intentioned, should take into account any negative effects, including a potential increase in malaria risk (invoking the Hippocratic dictum “first, do no harm”). In Malawi, replacing wood or charcoal fuels with cleaner burning “biomass” stoves, intended to reduce household-acquired respiratory illness by reducing domiciliary air pollution, was associated with significantly increased malaria infection rates in children occupying those houses. ^5^ It is unfortunately common for social programs implemented to improve living conditions related to food, like these, to actually worsen malaria. For example, installation of water service lines and catchments in Kakuma refugee camp in northwestern Kenya, for the purpose of providing water for gardening and for goat herds, created numerous larval habitats of *Anopheles arabiensis*, resulting in a malaria epidemic among the displaced persons residing there. ^6^ Similarly, intensified irrigation of rice in Malawi resulted in an increase in larval *Anopheles arabiensis* habitat, and higher indoor density of *Anopheles arabiensis* and malaria infection prevalence in households of farming families living near the rice plots compared with those farther away. ^7^^,^ ^8^ These actions, whether to improve air quality, food preparation, or food production, can have unintended but frankly quite predictable negative effects on malaria.

Second, elegant behavioral experimentation like that of Hennessee et al. ^2^ improves our understanding of how *Anopheles* mosquitoes respond to stimuli emanating from houses. In a recent timely study in Tanzania, poorly ventilated experimental huts with higher indoor temperatures and higher indoor CO_2_ concentrations attracted more *Anopheles arabiensis* than did well-ventilated huts with lower temperatures and lower CO_2_. ^9^ Perhaps, cooking with LPG increases CO_2_ concentrations (a strong attractant chemical), while not generating noxiously repellent smoke, possibly explaining the increased *Anopheles* hut entry and host-seeking observed by Hennessee et al. ^2^ With improved knowledge of the behavioral responses of *Anopheles* mosquitoes to human dwellings in sub-Saharan Africa, ^10^^,^ ^11^ inexpensive houses could be built that prevent mosquito entry yet incorporate such innovations as good ventilation and cooking with clean fuels, ^12^ resulting in a domicile in which it is safe to breathe and residents are safe from malaria. ^13^
